# Bioaccessibility and Antidiabetic Potential of *xique-xique* and *mandacaru* Fruits in a Simulated Gastrointestinal Tract Model

**DOI:** 10.3390/foods13203319

**Published:** 2024-10-18

**Authors:** Fábio Fernandes de Araújo, David de Paulo Farias, Iramaia Angélica Neri-Numa, Glaucia Maria Pastore, Alexandra Christine Helena Frankland Sawaya

**Affiliations:** 1 Faculty of Pharmaceutical Science, University of Campinas, UNICAMP, Campinas 13083-871, SP, Brazil; fabio.fernandesn18@gmail.com; 2 Bioflavors and Bioactive Compounds Laboratory, Department of Food Science, Faculty of Food Engineering, University of Campinas, Rua Monteiro Lobato, 80, Campinas CEP 13083-862, SP, Brazilglaupast@unicamp.br (G.M.P.)

**Keywords:** unconventional food plants, *Pilosocereus gounellei*, *Cereus jamacaru*, bioactive compounds, antioxidant potential, antihyperglycemic, antiglycation activity

## Abstract

This study evaluated the influence of gastrointestinal digestion on the bioaccessibility and antidiabetic potential of *xique-xique* (*Pilosocereus gounellei*) and *mandacaru* (*Cereus jamacaru*) fruits. After digestion, the content of total phenolics and flavonoids reduced by 58.3 and 73.51% in *xique-xique* and 48.33 and 88.43% in *mandacaru*. In addition, compounds such as rutin, ρ-coumaric acid, catechin and epicatechin reduced during digestion for both fruits. The antioxidant potential by the ABTS assay increased by 153.3% for *xique-xique* and 273.46% for *mandacaru* in the intestinal phase. However, using the ORAC assay, the antioxidant potential of *xique-xique* reduced from 255.42 to 112.17 μmol TE g^−1^. The capacity of *xique-xique* fruit to reduce α-amylase activity reduced 23.71-fold after digestion, but the potential to inhibit α-glucosidase increased 17.8-fold. The antiglycation potential reduced in both fruits after the in vitro gastrointestinal digestion. Thus, the bioaccessibility of the phenolic compounds from the fruits, as well as their functional potential, were influenced by the digestive process, as well as by the sample evaluated.

## 1. Introduction

The World Health Organization reports that chronic non-communicable diseases (NCDs), including heart disease, cancer, and diabetes, represent a significant public health challenge, being responsible for approximately 74% of all deaths globally [1]. Diabetes has become a significant global concern, as the International Diabetes Federation (IDF) reports that 1 in 11 adults is affected by this disease, amounting to approximately 463 million people. Projections for 2045 estimate that this figure could rise to 700 million. Additionally, it is important to note that about 10% of total health expenditures, approximately USD 760 billion, is currently allocated to the treatment of diabetes [2].

Type 2 diabetes mellitus is a metabolic condition marked by elevated blood sugar levels. This occurs either due to decreased insulin sensitivity or a deficiency in insulin signaling. These issues can result in complications affecting various organs and tissues, including the nerves, eyes, kidneys, and cardiovascular system [3]. Scientific evidence suggests that consuming fruits and vegetables rich in bioactive compounds, such as polyphenols, may be a promising approach for the treatment and prevention of diseases like diabetes [3,4].

Phenolic compounds are secondary metabolites produced by plants that have garnered significant interest from the scientific community for their health benefits. These effects are largely attributed to their ability to sequester or inhibit reactive species, donate electrons to free radicals, and activate antioxidant enzymes, among other mechanisms [3,5,6]. Consuming these compounds in sufficient amounts may offer a range of beneficial effects in preventing various diseases, including diabetes, cancer, obesity, Parkinson’s disease, Alzheimer’s disease, and cardiovascular and intestinal disorders, among others [5]. While numerous health benefits are associated with the consumption of these compounds, their functional and bioactive properties can be influenced by various factors, particularly their bioaccessibility following gastrointestinal digestion. Bioaccessibility refers to the amount of a specific food component that is released and solubilized in the intestinal lumen during digestion, making it available for absorption. This process is affected by several factors, including the concentrations of substances in food, their release from the food matrix, chemical structure, molecular interactions with other compounds, molecular size, degree of polymerization, and solubility etc. [3,5,6,7,8].

Cactus from the Cactaceae family, including *Pilosocereus gounellei* (F.A.C. Weber) and *Cereus jamacaru* DC, commonly known as *xique-xique* and *mandacaru*, play a vital role in the Brazilian semi-arid region [9]. These species have excellent nutritional value, being considered a good source of nutrients such as carbohydrates, fiber and minerals [10]. Due to their richness in nutrients, previous research has demonstrated that these species have significant industrial potential for the development of various products, including cakes, ice creams, yogurts, and juices [11,12,13]. Additionally, recent studies have revealed that the fruits of species like *xique-xique* and *mandacaru* are rich sources of various bioactive compounds, including phenolic compounds such as chlorogenic acid, catechin, epicatechin, myricetin, quercetin, kaempferol, rutin, and others [14,15]. Furthermore, a recent review paper highlighted that these species may offer numerous health benefits for consumers, exhibiting properties such as antioxidant, in addition to anti-inflammatory, anti-obesity, anti-diabetic, anti-cancer, and anti-nociceptive effects [16]. However, so far, information regarding the effect of the in vitro gastrointestinal digestion process on the bioactivity and bioaccessibility of the phenolic compounds present in these fruits is still scarce.

Studying the effects of the digestive process is important to understand the interactions that may affect their nutritional potential and, therefore, developing strategies to increase their biological effects is essential to encourage the use of underutilized species such as *xique-xique* and *mandacaru* for the preparation of new foods with functional claims. Thus, the objective of this study was to investigate the influences of in vitro gastrointestinal digestion on the profile of phenolic compounds, antioxidant potential, α-amylase and α-glucosidase inhibitory activities and antiglycation properties of *xique-xique* and *mandacaru* fruits.

## 2. Material and Methods

### 2.1. Sample Acquisition and Preparation

*xique-xique* and *mandacaru* fruit were collected in the city of Solânea in the interior of Paraíba, Brazil. The botanical identification of the species was carried out, the exsiccates (Accession nº 208.839 and 208.840) were deposited in the Herbarium-UEC of the Institute of Biology of the State University of Campinas UEC-UNICAMP and the project was registered in the National System for the Management of Genetic Knowledge and Traditional Associate –SisGen (N° ABFC0D8). The collected whole fruits were washed with distilled water and processed in a home juicer. Immediately after processing, they were immediately frozen (−20 °C), freeze-dried and ground in a knife mill. The samples thus obtained were placed in dark packaging and stored at −20 °C to avoid possible changes in chemical composition.

### 2.2. In Vitro Gastrointestinal Digestion

The digestion process was simulated following the methodology described by Sancho and collaborators [17]. Briefly, 500 mg of each fruit (freeze-dried whole fruits) was homogenized in 9 mL of saline solution (140 mmol L^−1^ NaCl, 5 mmol L^−1^ KCl) and the pH of the mixture was adjusted to 2.0 with 6 mol L^−1^ HCl. An aliquot of 125 μL of porcine pepsin solution (Sigma-Aldrich, 200 mg of 424 U mg^−1^ pepsin in 5 mL of 0.1 mol L^−1^ HCl) was added and the samples were incubated in a water bath with shaking at 130 rpm for 1 h. A sample of 2 mL of this phase was collected and stored in a freezer at −80 °C for later analysis.

After the gastric digestion steps, the pH of the samples was adjusted again to 6.8 by adding 1 mol L^−1^ NaHCO_3_. Then, 625 μL of pancreatin and bile solution (Sigma-Aldrich, 225 mg of bile extract and 37 mg of pancreatin with activity equivalent to 4x U.S.P, diluted in 18.7 mL of NaHCO_3_ 0.1 mol L^−1^) were added and the samples were incubated at 37 °C and 130 rpm for 2 h. All fractions were ultrafiltered using Amicon ultra centrifugal filtration devices (30 kDa, Millipore) and stored at −80 °C until analysis. A sample of saline solution at pH 7.0 (blank) was also subjected to all digestion procedures to eliminate reagent interference. The samples collected after each phase, as well as the undigested samples (suspended in water like a juice), were filtered and used as extracts to perform all analyses.

### 2.3. Quantification of Phenolic Compounds

The identification and quantification of phenolic compounds were performed in Selected Ion Monitoring (SIM) mode (Figures S1–S3—Supplementary Materials) using an Acquity UHPLC chromatograph coupled to an Acquity TQD mass spectrometer (Micromass-Waters Manchester, Manchester, UK) with an ESI source, Waters Acquity C18 BEH column (2.1 mm × 50 mm × 1.7 µm), following the procedures described by Cassola and colleagues [18]. For this analysis, a specific chromatographic method for the samples was developed using the following conditions: phase A composed of acidic water (0.1% formic acid) and phase B of acetonitrile (HPLC grade). The gradient started and remained at 95% A and 5% B for 1 min, then increased to 100% B for 8 min and returned to the initial conditions for 1 min, totaling 10 min. The flow rate was 0.2 mL/min and the injection volume was 7 µL. The results were obtained through calibration curves (concentrations ranging from 0.005 to 100 µg mL^−1^) constructed with standard compounds of quinic acid, rutin, p-coumaric acid, catechin and epicatechin from Sigma-Aldrich and the values were expressed in mg 100 g^−1^ of lyophilized sample (fd).

### 2.4. Determination of Total Phenolic Content

This assay was conducted following the method proposed by Singleton et al., with minor modifications [19]. In brief, 30 µL of fruit extracts was mixed with 150 µL of 10% Folin–Ciocalteau reagent and 120 µL of 7.5% NaHCO_3_, then incubated for 6 min at 45 °C. The absorbance was measured at 760 nm, and the total phenolic content in the samples was quantified using a standard curve created with gallic acid, with results expressed as mg of gallic acid equivalents (GAE) g^−1^ fd.

### 2.5. Determination of Total Flavonoid Content

The total flavonoid content was determined using the method established by Zhishen et al. [20]. For the reaction system, 30 μL of fruit extracts was combined with 110 μL of ultrapure water. Next, 8 μL of 5% NaNO_2_ was added, and after 5 min, 8 μL of 10% AlCl_3_ was introduced, followed by an additional 6 min incubation. Finally, 50 μL of 1 M NaOH and 70 μL of ultrapure water were added. The absorbance of the mixture was measured at 510 nm, with catechin used to prepare the analytical curve; the total flavonoid content was expressed as mg of catechin equivalents (EC) g^−1^ fd.

### 2.6. ABTS Assay

The antioxidant capacity was assessed using the ABTS+ assay, which involved reacting 5 mL of ABTS (7 mM) with 88 µL of 140 mM potassium persulfate [21]. After the formation of the ABTS radical, it was diluted with distilled water to achieve an absorbance value of 0.700 ± 0.02 at 734 nm using a microplate reader. Trolox was employed to prepare the analytical curve, and the results were expressed in µM Trolox equivalents (TE) g^−1^ fd.

### 2.7. ORAC Assay

This assay was performed according to Prior et al., with minor modifications [22]. Samples, standards and reagents were prepared in 75 mM potassium phosphate buffer, pH 7.4. The reaction system consisted of 20 μL of Trolox standard or previously diluted extracts, 120 μL of fluorescein (0.378 μg mL−1, pH 7.4) and 60 μL of AAPH [2,2′-Azobis(2-methylpropionamidine) dihydrochloride] (108 mg/mL). Fluorescence intensity was monitored at 37 °C immediately after the addition of AAPH every 60 s cycle for 80 cycles in a NOVOstar Microplate Reader. Analyses were performed in triplicate and results were expressed in µM TE g^−1^ fd.

### 2.8. Antidiabetic Potential

#### 2.8.1. α-Amylase Activity

The inhibition of α-amylase activity was evaluated according to the methodology described by Van Quan et al., with modifications [23]. To this end, 20 μL of each extract was pre-incubated for 10 min at 25 °C with 20 μL of 1 U/mL porcine pancreatic α-amylase solution dissolved in 0.1 M sodium phosphate buffer (pH 6.9) containing 0.006 M sodium chloride. Then, 30 μL of starch solution (0.25%) was added and incubated again for 6 min at 25 °C. To stop the reaction, 20 μL of hydrochloric acid (1 M) was pipetted. Next, 120 μL of Lugol’s aqueous solution (250 μL of 5% solution) was added to develop a dark blue color. The absorbance was read at 565 nm in a microplate reader. Acarbose was used as a positive control and the inhibitory activity was expressed as IC_50_.

#### 2.8.2. α-Glucosidase Activity

The inhibition of α-glucosidase activity was evaluated according to Van Quan and collaborators [23]. Briefly, an amount of 20 μL of each extract was mixed with 20 μL of 0.1 M potassium phosphate buffer (pH 7) and 40 μL of α-glucosidase (from Saccharomyces cerevisiae, Sigma-Aldrich, St Louis, MO, USA) (0.5 U/mL in 0.1 M potassium phosphate buffer, pH 7). After 6 min of incubation at 25 °C, a 20 μL aliquot of 5 mM ρ-nitrophenyl-α-D-glucopyranoside substrate (in 0.1 M potassium phosphate buffer, pH 7) was added and the mixture was incubated for another 8 min. The reaction was terminated by the addition of 100 μL of 0.2 M Na_2_CO_3_, and absorbance was recorded at 405 nm. Acarbose was used as a positive control. The inhibitory activity of the extracts on α-glucosidase was calculated as the percentage of inhibition and the results were expressed as IC_50_ (mg mL^−1^).

#### 2.8.3. Antiglycation Potential

The antiglycation potential of the extracts was determined according to the protocol described by Sri Harsha et al., with some modifications [24]. Briefly, 200 mM potassium phosphate buffer (pH 7.4) containing 0.02% sodium azide, 50 mg/mL BSA in phosphate buffer, was mixed with 1.25 M fructose in phosphate buffer and the extract. The control consisted of phosphate buffer instead of the antiglycation agent and for the blank, fructose and the antiglycation agent were replaced by phosphate buffer. The mixtures were incubated at 37 °C for 3 days in a water bath in the absence of light. The fluorescence of the mixture was measured in a microplate reader at an excitation/emission wavelength of 350/420 nm, respectively and the results were calculated as percentage of inhibition and expressed as IC_50_ (mg mL^−1^).

### 2.9. Statistical Analysis

All experiments conducted in this study were performed in triplicate, and the results were reported as means ± standard deviations. The values of the tested variables were analyzed using analysis of variance (ANOVA), and when significant differences were found, the means of the treatments were compared using Tukey’s and Student’s *t* tests, with a significance level of *p* ≤ 0.05. Statistical analyses were carried out using Statistica software version 7.0 [25].

## 3. Results and Discussion

Table 1 shows the total phenolic and total flavonoid content before and during the in vitro gastrointestinal digestion process. The lowest content of total phenolic compounds in the undigested samples was observed for *xique-xique* (11.87 ± 0.67 mg GAE g^−1^ fd), being approximately 1.48 times lower than the value observed for *mandacaru* (17.63 ± 0.08 mg GAE g^−1^ fd). In addition, the greatest reduction in the content of total phenolics in the intestinal phase was also observed in *xique-xique*, which presented a reduction of 58.3%, while the reduction for *mandacaru* was 48.33%. Likewise, flavonoid content was greatly reduced after gastrointestinal digestion. The highest total flavonoid content (16.78 mg CE g^−1^ df) was found in the undigested sample for *mandacaru* (*p* ≤ 0.05); however its content reduced approximately 24.97 and 88.43% in the gastric and intestinal phases, respectively. Similarly, the total flavonoid content also reduced approximately 2.97% (gastric phase) and 73.51% (intestinal phase) in *xique-xique* after the in vitro gastrointestinal digestion process.

So far, few studies have sought to evaluate the effect of the gastrointestinal digestive process in cactus species, this being the first carried out with *xique-xique* and *mandacaru* fruits. Similar results to those found in this study were reported by Santiago and colleagues when they evaluated the influence of the gastrointestinal digestion process on the content of bioactive compounds in cladodes of *Opuntia ficus-indica*, a species of cactus belonging to the same family as *xique-xique* and *mandacaru*. According to the authors, the content of total flavonoid compounds decreased from 120 ± 0.49 (before digestion) to 58.30 ± 2.28 in the gastric phase and 44.96 ± 0.89 mg g^−1^ dry matter (dm) in the intestinal phase, while the content of total phenolic compounds and total phenolic acids decreased by approximately 55.90 and 44.49%, respectively [26]. In another study, a small reduction in the content of total phenolic and flavonoid compounds was observed after the digestive process in yogurt containing 10% cactus pear extract (*Opuntia oligacantha* C.F. Först). The reported values of total phenolic compounds were 19.9: 17.5 and 16.4 mg GAE 100 mL^−1^ for the undigested samples, gastric and intestinal phases, respectively, representing a reduction of approximately 12.06% in the intestinal phase. Similarly, the reduction in the content of flavonoids was 2.79%, with the reported values being 17.9, 18.2 and 17.4 mg QE 100 mL^−1^ for the undigested samples, gastric and intestinal phases, respectively. [27]. Different results were observed for oat-wheat bread enriched with red *pitaya* powder (*Hylocereus polyrhizus*), where the content of total phenolic compounds increased by about 5 and 15% (2.75–3.93 mg GAE g^−1^) after intestinal digestion [28].

The behavior of the content of total phenolic compounds and total flavonoids was similar to that observed for the profile of individual phenolic compounds after the digestive process for both fruits (Table 2). There was a reduction in the content of phenolic compounds such as rutin, ρ-coumaric acid, catechin and epicatechin in the gastric and intestinal phases for *xique-xique*, with the exception of quinic acid, which demonstrated an increase in its content after the digestive process (1.18-fold in the gastric phase and 1.2 in the intestinal phase). A lower content of individual phenolic compounds was observed in *mandacaru* fruit and, unlike the behavior observed in *xique-xique* fruit, the quinic acid content reduced after the digestive process (reduction of 61.78% in the gastric phase and 49.10% in the intestinal phase). In the gastric phase, a content of 1.40 ± 0.01 mg 100 g^−1^ of catechin was observed for *mandacaru*; however, this value reduced in the intestinal phase.

The release of phenolic compounds during the digestive process may vary depending on the sample studied and the bioactive compounds present in the matrix. In whole fruit extracts of cactus berry (*Myrtillocactus geometrizans*), for example, a reduction in the content of some phenolic compounds such as epigallocatechin-glucoside, quercetin-3-*O*-rhamnosyl rutinoside-glucoside, quercetin-3-*O*-rhamnosyl-rhamnosyl-rhamnosyl-glucoside, quercetin-3-*O*-neohesperidosyl-rhamnoside, quercetin-3-rutinoside, and kaempferol-7-*O*-neohesperidoside, among others, was observed [4]. Saniovanni and colleagues, when studying the effect of gastrointestinal digestion on the phenolic compounds of *Vitis vinifera* L. leaves, observed that there was a reduction in the content of several phenolic compounds, including caffeic acid derivatives, flavonols and anthocyanins. In their study, the rutin content, for example, reduced from 1.31 to 1.15 in the gastric phase and 0.96 mg g^−1^ in the intestinal phase. Other compounds such as caftaric acid, quercetin 3-*O*-glucoside, kaempferol 3-*O*-glucoside and delphinidin 3-*O*-glucoside showed a reduction of around 20.02; 38.19; 38.72 and 71.57%, respectively [29]. Similar behavior was also verified in blueberry phenolic compounds after the digestive process, wherein a reduction in the content of flavonoids such as quercetin arabinoside (0.113 to 0.084 mg g^−1^) and anthocyanins such as delphinidin 3-galactoside (0.107 to 0.004 mg g^−1^) and malvidin 3-galactoside (0.270 to 0.025 mg g^−1^) was observed in the intestinal phase; however, the chlorogenic acid content increased by around 18.55%, from 0.291 to 0.345 mg g^−1^ [30]. Compounds such as epicatechin and gallic acid present in wild blackberry fruits reduced by approximately 99.05 and 66.05%, while other compounds such as catechin, quercetin and kaempferol reduced completely in the intestinal phase. On the other hand, some phenolic acids including *p*-coumaric acid and ferulic acid increased their content after the digestive process from 14.97 to 192.00 and 6.95 to 92.67 mg mL^−1^, respectively [31].

The antioxidant capacity of fruit during the digestive process can be found in Table 1. It can be seen that the antioxidant capacity in the ABTS assay increased for both samples after gastrointestinal digestion. In *xique-xique,* the increase was 47.78% in the gastric phase and 153.3% in the intestinal phase. In *mandacaru,* the increase was 160.55% and 273.46% in the gastric and intestinal phases, respectively. However, statistical analysis demonstrated that there is no difference between the intestinal-phase samples of both fruit (*p* ≥ 0.05).

On the other hand, the antioxidant capacity in the ORAC assay significantly reduced during the digestive process in the *xique-xique* sample, with values reducing from 255.42 ± 11.05 to 104.43 ± 5.85 in the gastric phase and 112.17 ± 14.35 μmol TE g ^−1^ in the intestinal phase. In *mandacaru*, a reduction in antioxidant capacity was also observed for this assay, although without statistical difference between the three phases evaluated. As can be seen, the behavior of the antioxidant potential of the samples varied depending on the assay used. This behavior may be related to the reduction of some phenolic compounds and to the release of others that could be bound to the cellular matrix and that were released after the digestive process. Phenolic compounds are recognized as natural antioxidants and anti-inflammatory agents, and their beneficial effects are due to their potential to sequester or inhibit reactive species, inactivate free radicals and activate antioxidant enzymes, acting in the prevention and treatment of various chronic non-communicable diseases [5].

The effect of the digestive process on the antioxidant activity of cactus, as well as other native Brazilian fruits, has also been reported. For example, for the ABTS assay, a reduction in the antioxidant potential in raw and cooked cladodes of *Opuntia ficus-indica* was observed after in vitro gastrointestinal digestion [26]. On the other hand, the digestive process resulted in an increase in the antioxidant potential determined by the DPPH and FRAP tests in the gastric and intestinal phases in the oat-wheat bread fortified with red *pitaya* powder [28]. Araujo and colleagues, when studying the effect of in vitro gastrointestinal digestion on *araça-boi* pulp (*Eugenia stipitata*), reported that the antioxidant capacity measured by the DPPH assay reduced from 8.4 to 0.3 μmol TE g^−1^ dw in the intestinal phase, while for the ABTS and ORAC assays an increase of approximately 2.78 and 3.19 times was observed, respectively, after the digestive process [6]. According to the authors, after the digestive process there was a reduction in phenolic acids and a greater release of flavonoids, such as apigenin caffeate hexoside, kaempferol diacetyl dicoumaroylhexoside, quercetin hexopyranosylhexoside and others, directly impacting the increase in antioxidant potential determined by ABTS and ORAC tests [6]. Similarly, the antioxidant capacity in the pulp of *uvaia* (*Eugenia pyriformis*) fruit also increased after gastrointestinal digestion for the ABTS and ORAC assays, with values for ABTS increasing from 44.81 to 77.13 μmol TE g^−1^ dm [7]. In wild blackberry fruit a reduction in the antioxidant potential of the samples occurred during the in vitro gastrointestinal digestion process. In that study, it was possible to observe a reduction of approximately 52.43 and 40.47% in the intestinal phase for the ORAC and ABTS assays, respectively [31]. Similar to the results reported in the present study, there was an increase of approximately 3.06- and 17.32-fold in the antioxidant potential measured by the ABTS assay for hulled barley, after the digestion process [32].

The results regarding the activities of digestive enzymes before and after the digestive process can be seen in Table 3; the IC_50_ of α-amylase for *xique-xique* increased after the gastrointestinal digestion process, and thus, in the intestinal phase, the potential to inhibit this enzyme was reduced by 23.71-fold for this fruit. On the other hand, the potential to inhibit α-amylase increased in *mandacaru* after the digestive process, since the IC_50_ of the sample was reduced in the intestinal phase (77.63 to 46.22). Only undigested samples and the gastric phase of *xique-xique* showed similar potential to arcabose (the therapeutic agent used as a standard to evaluate the inhibition of digestive enzymes) (*p* > 0.05). Regarding the activity of α-glucosidase, except for the undigested *xique-xique* sample, all other samples showed greater potential to inhibit the enzyme when compared to arcabose. Furthermore, there was a 17.8- and 1.68-fold increase in the inhibition potential of α-glucosidase in *xique-xique* and *mandacaru* fruits in the intestinal phase. As previously reported, this increase in the inhibitory potential of digestive enzymes, as well as in the antioxidant potential in the ABTS assay, may be related to the release of bioactive compounds linked to the fiber content or other nutrients present in the fruits. According to Fend and Kong, phenolic compounds are compounds with antioxidant properties that can act competitively or even non-competitively in the inhibition of digestive enzymes, such as α-amylase and α-glucosidase [33].

Recently, the inhibition of enzymes such as α-amylase and α-glucosidase has been identified as a good strategy for controlling hyperglycemia and treating diabetes, since the inhibition of these enzymes is essential to reduce carbohydrate hydrolysis and glucose uptake and to favor the control of postprandial hyperglycemia [3,34]. However, to date, there is little information in the literature regarding the effect of gastrointestinal digestion in cactus on the inhibition of digestive enzymes, as well as on the inhibition of protein glycation. For this reason, our study is extremely important, since it stands out as one of the first to report the antidiabetic potential of species belonging to the Cactaceae family after the digestive process.

Likewise, when compared to the results observed in our study, α-amylase inhibition increased significantly at a concentration of 150 μg mL^−1^ of *Inonotus obliquus* samples after gastric digestion, with the highest inhibitory activity for this enzyme (37.96%) being observed at a concentration of 200 μg mL^−1^. Similarly, the inhibitory activity of α-glucosidase also increased significantly in the gastric and intestinal phases with the IC_50_ of 60.24 μg mL^−1^ and 52.97 μg mL^−1^, respectively [35]. In water-soluble polysaccharides from *Opilia amentacea* Roxb fruit, a 69% of α-amylase inhibition in the pre-digested sample at a concentration of 50 μg mL^−1^ was observed, whereas the values of the pancreatic and gastric sample were 72 and 49%, respectively. For α-glucosidase, an inhibition of 70% was observed in the gastric-digested sample and 65% in the pre-digested sample [36]. Different results were found by Gutiérrez and collaborators, depending on the species of oregano studied. For *Hedeoma patens*, for example, the inhibition of α-amylase activity increased from 23.93% in the undigested sample to 25.43 and 52.53% in the gastric and intestinal phases, respectively. On the other hand, there was a reduction in the inhibition of α-glucosidase of approximately 4.80-fold in the gastric phase and 6.68-fold in the intestinal phase. In this study, the authors also observed that certain phenolic compounds disappeared, while others were only found after the digestive process, which certainly influenced the potential of the samples to inhibit digestive enzymes [37].

High blood sugar levels can result in the glycation of proteins and, consequently, the formation and accumulation of advanced glycation end products (AGEs). This process occurs through the binding of carbonyl groups of sugars such as glucose, fructose and ribose to free amino groups of proteins. AGES, in turn, are carbonyl compounds originating from various chemical reactions in Schiff base intermediates that are converted into more stable Amadori products. These can cause changes in proteins, lipids and DNA, resulting in several problems in diabetic patients such as neuropathy, nephropathy, etc. [3,38,39]. Therefore, the search for new natural agents capable of inhibiting protein glycation may be a promising alternative to reduce some diabetic complications [40].

**Figure 1:**
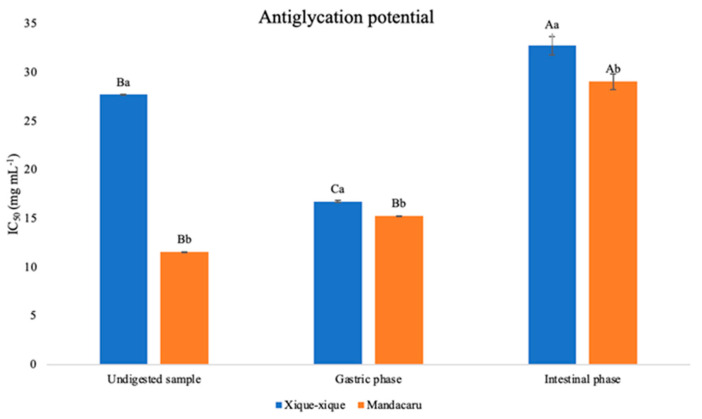
Antiglycation potential of *xique-xique* and *mandacaru* fruit after in vitro gastrointestinal digestion. Capital letters indicate statistical difference for each treatment in the different digestive phases determined by Tukey’s test at 5% significance (*p* ≤ 0.05). Lower case letters indicate statistical difference between treatments within the same digestive phase determined by *t* test at 5% significance (*p* ≤ 0.05).

As can be seen in Figure 1, the undigested sample from *mandacaru* fruit showed a higher antiglycation potential (IC_50_ 11.59 ± 0.001 mg mL^−1^) than the extract obtained from *xique-xique* fruits (IC_50_ 27.72 ± 0.01 mg mL^−1^). After the in vitro gastrointestinal digestion process, it was possible to observe a reduction in the antiglycation potential for both samples in the intestinal phase, with *mandacaru* reducing its potential by approximately 2.50-fold, presenting an IC_50_ of 29.07 ± 0.80 mg mL^−1^. Despite there being a reduction in antiglycation potential of *xique-xique* in the intestinal phase (27.72 to 32.40 mg mL^−1^), there was an increase of approximately 1.65-fold in the gastric phase. These results demonstrate that the antiglycation potential depends on the fruit and the phase studied. Contrary to what was observed for the antioxidant capacity and the potential to inhibit digestive enzymes, the reduction in the antiglycation potential appears to be related to the reduction in the content of phenolic compounds, total flavonoids and compounds such as rutin. Rutin is a flavonol found naturally in plant species that has great bioactive potential. A study previously carried out by Dubey et al. demonstrated that rutin at a concentration of 33 mg ml -1 presented antiglycation potential, being able to inhibit 35.55% of fructosamine glycation, 13.45% of protein carbonyls and 80.27% of protein thiols [41]. Thus, the reduction in the concentration of this compound, as well as other phenolic compounds, certainly impacted the antiglycation capacity of the *xique-xique* and *mandacaru* samples after the digestive process.

Different results from ours were observed for the berries of *E. umbellata* and *S. lanceolata*, which showed an increase of 1.74- and 1.85-fold, respectively, after the digestion process [38]. On the other hand, the antiglycation activity of *Vaccinium myrtillus* fruit extract reduced by approximately 26% after gastrointestinal digestion, since the IC_50_ went from 70.41 mg L^−1^ in the undigested sample to 75.15 and 89.04 mg L^−1^ in the gastric and intestinal phases, respectively [42]. Likewise, the BSA glycation potential of phenolic compounds from jabuticaba (*Plinia cauliflora*) peel reduced approximately 4.27-fold after the digestive process. In that study, the IC_50_ went from 0.37 in the undigested sample to 0.59 and 1.58 mg mL^−1^ for the gastric and intestinal phases, respectively [43].

## 4. Conclusions

In this study, we determined for the first time the bioaccessibility of phenolic compounds from *xique-xique* and *mandacaru* fruits, as well as their antidiabetic properties during the in vitro gastrointestinal digestion process. The content of total phenolics and total flavonoids in *xique-xique* and *mandacaru* fruit reduced after the digestion process. Similarly, there was also a reduction in the content of phenolic compounds such as rutin, ρ-coumaric acid, catechin and epicatechin for both fruits, except for quinic acid, which presented an increase in its content after the digestive process in *xique-xique* fruit. Despite these results, other phenolic compounds may have been released from the plant matrices during the digestion process, which could certainly have influenced the increase in antioxidant potential in the ABTS assay for both fruits and in the ORAC assay for *mandacaru*. Likewise, the antihyperglycemic potential of the samples was positively affected after the digestive process, except for *xique-xique*, which reduced its potential to inhibit α-amylase in the intestinal phase. On the other hand, the antiglycation potential for both samples were reduced after the in vitro gastrointestinal digestion process. Therefore, the functional and antidiabetic potential of the samples depended on the matrix and digestive phase evaluated.

Finally, the results of this study may provide important insights into the impact of the digestion process on the bioactive properties of underutilized fruits from the semi-arid region of Brazil, as well as facilitating their investigation and application in the development of foods with functional claims that may contribute to the maintenance of health.

## Figures and Tables

**Table 1:** Total phenolics, total flavonoids and antioxidant capacity of *xique-xique* and *mandacaru* fruit after in vitro gastrointestinal digestion.

Fruit	Treatment	Total Phenolic ^1^	Total Flavonoids ^2^	ABTS ^3^	ORAC ^3^
*xique-xique*	Undigested sample	11.87 ± 0.67 Ab	9.25 ± 0.12 Ab	58.64 ± 2.53 Ca	255.42 ± 11.05 Aa
	Gastric phase	9.61 ± 0.39 Bb	8.05 ± 0.16 Bb	86.66 ± 0.99 Bb	104.43 ± 5.85 Bb
	Intestinal phase	4.95 ± 0.24 Cb	2.45 ± 0.05 Ca	148.55 ± 0.91 Aa	112.17 ± 14.35 Bb
*mandacaru*	Undigested sample	17.63 ± 0.08 Aa	16.78 ± 0.42 Aa	39.80 ± 2.87 Cb	172.28 ± 1.31 Bb
	Gastric phase	17.38 ± 0.04 Aa	12.59 ± 0.16 Ba	103.70 ± 1.70 Ba	168.63 ± 2.04 Ba
	Intestinal phase	9.11 ± 0.52 Ba	1.94 ± 0.04 Cb	148.64 ± 0.48 Aa	174.53 ± 1.73 Aa

Capital letters indicate statistical difference for each treatment in the different digestive phases determined by Tukey’s test at 5% significance (*p* ≤ 0.05). Lower case letters indicate statistical difference between treatments within the same digestive phase determined by *t* test at 5% significance (*p* ≤ 0.05). ^1^ Results expressed as mg GAE g^−1^ fd. ^2^ Results expressed as mg CE g^−1^ fd. ^3^ Results expressed as µM TE g^−1^ fd.

**Table 2:** Phenolic compounds identified and quantified in *xique-xique* and *mandacaru* fruit before and after in vitro gastrointestinal digestion.

Fruit	Treatment	Compound				
		Quinic Acid	Rutin	ρ-Coumaric Acid	Catechin	Epicatechin
*xique-xique*	Undigested sample	1.90 ± 0.01 Bb	9.27 ± 0.27 Aa	0.34 ± 0.09 Ab	n.d.	n.d.
	Gastric phase	2.26 ± 0.11 Ab	0.19 ± 0.07 Ba	n.d.	n.d.	0.44 ± 0.0001
	Intestinal phase	2.29 ± 0.08 Ab	n.d.	n.d.	0.49 ± 0.006	n.d.
*mandacaru*	Undigested sample	5.60 ± 0.08 Aa	0.10 ± 0.007 Ab	0.67 ± 0.03 Aa	n.d.	n.d.
	Gastric phase	3.46 ± 0.04 Ba	0.0024 ± 0.0002 Bb	0.23 ± 0.03 B	1.40 ± 0.01	n.d.
	Intestinal phase	2.75 ± 0.34 Ca	n.d.	n.d.	n.d.	n.d.

Results expressed as mg 100 g^−1^ fd. Capital letters indicate statistical difference for each treatment in the different digestive phases determined by Tukey’s test at 5% significance (*p* ≤ 0.05). Lower case letters indicate statistical difference between treatments within the same digestive phase determined by *t* test at 5% significance (*p* ≤ 0.05). “n.d.”, not detected.

**Table 3:** Digestive enzyme inhibition potential of *xique-xique* and *mandacaru* fruit before and after in in vitro gastrointestinal digestion.

Enzyme	Fruit	Treatment	IC_50_ (mg mL^−1^)
α-amylase	*xique-xique*	Undigested sample	30.91 ± 1.03 Eb
		Gastric phase	33.68 ± 3.10 Eb
		Intestinal phase	733.15 ± 93.47 Ba
	*mandacaru*	Undigested sample	77.63 ± 9.61 Ca
		Gastric phase	1372.54 ± 107.83 Aa
		Intestinal phase	46.22 ± 3.39 Db
	-	Acarbose	36.60 ± 2.80 E
α-glycosidase	*xique-xique*	Undigested sample	160.92 ± 3.13 Aa
		Gastric phase	29.92 ± 2.70 Da
		Intestinal phase	9.04 ± 0.24 Fa
	*mandacaru*	Undigested sample	41.81± 0.55 Cb
		Gastric phase	18.75 ± 0.30 Eb
		Intestinal phase	8.22 ± 0.04 Fa
	-	Acarbose	121.79 ± 2.78 B

Capital letters indicate statistical difference between treatments in different digestive phases determined by Tukey’s test at 5% significance (*p* ≤ 0.05). Lower case letters indicate statistical difference within each treatment in specific digestive phases determined by Tukey’s test at 5% significance (*p* ≤ 0.05).

## Data Availability

The original contributions presented in the study are included in the article/Supplementary Materials, further inquiries can be directed to the corresponding author.

## Supplementary Materials

The following supporting information can be downloaded at: https://www.mdpi.com/article/10.3390/foods13203319/s1, Figure S1. Chromatogram of xique-xique and mandacaru undigested samples in LC/MS analysis in Selected Ion Monitoring (SIM) mode; Figure S2. Chromatogram of samples from the gastric phases of xique-xique and mandacaru in LC/MS analysis in Selected Ion Monitoring (SIM) mode; Figure S3. Chromatogram of samples from the intestinal phases of xique-xique and mandacaru in LC/MS analysis in Selected Ion Monitoring (SIM) mode.
