# Supplementary material for: Bioaccessibility and Antidiabetic Potential of xique-xique and mandacaru Fruits in a Simulated Gastrointestinal Tract Model
Source: Foods. 2024 Oct 18;13(20):3319. doi: 10.3390/foods13203319 (PMC11507249; doi:10.3390/foods13203319)
Supplement: Supplementary file 1 [file foods-13-03319-s001.zip › foods-3237293-supplementary.pdf]

Supplementary material

Figure S1. Chromatogram of *xique-xique* and *mandacaru* undigested samples in LC/MS analysis in Selected Ion Monitoring (SIM) mode.

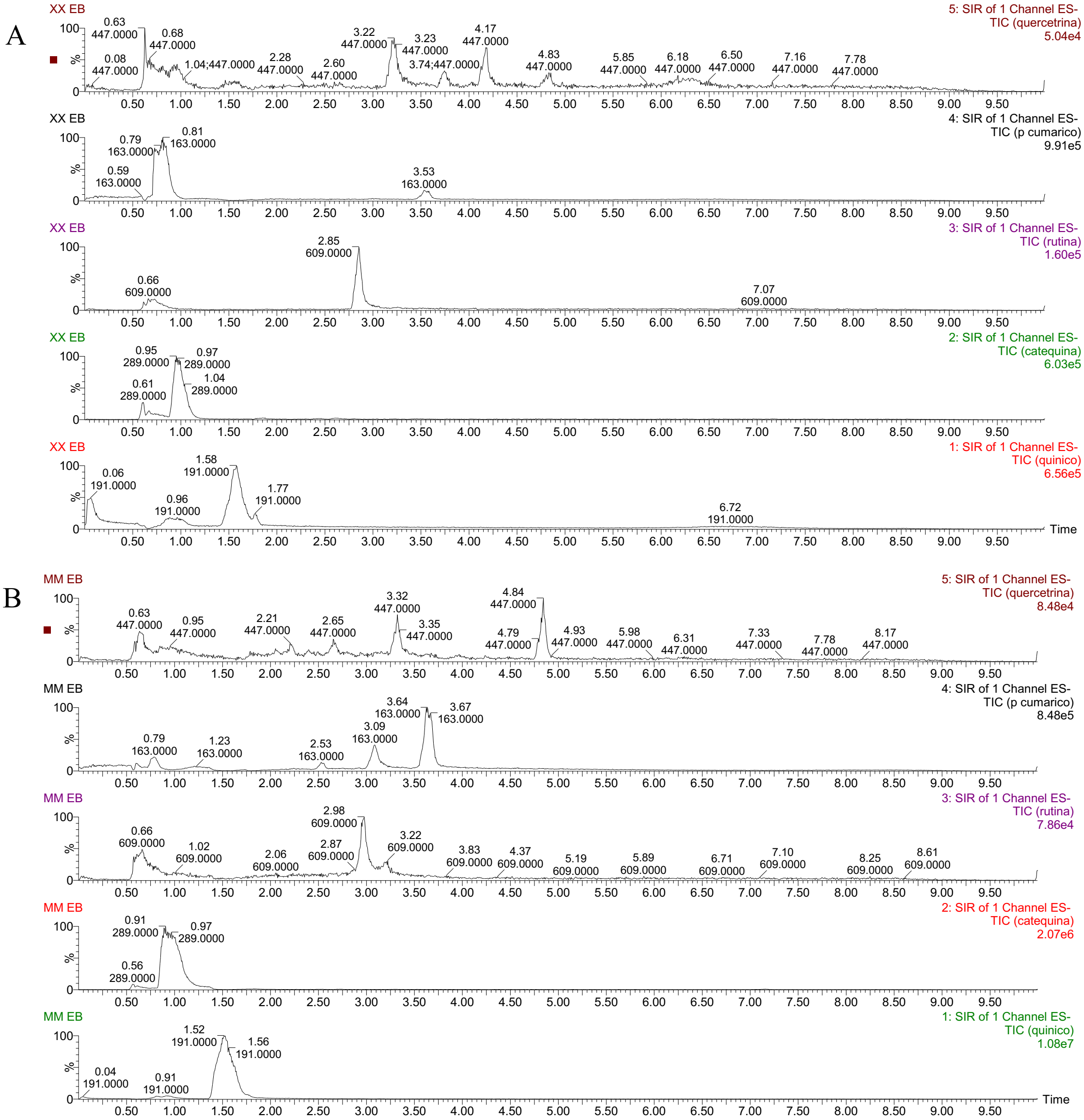

Undigested *xique-xique* fruits (A); Undigested *mandacaru* fruits (B)

Figure S2. Chromatogram of samples from the gastric phases of *xique-xique* and *mandacaru* in LC/MS analysis in Selected Ion Monitoring (SIM) mode.

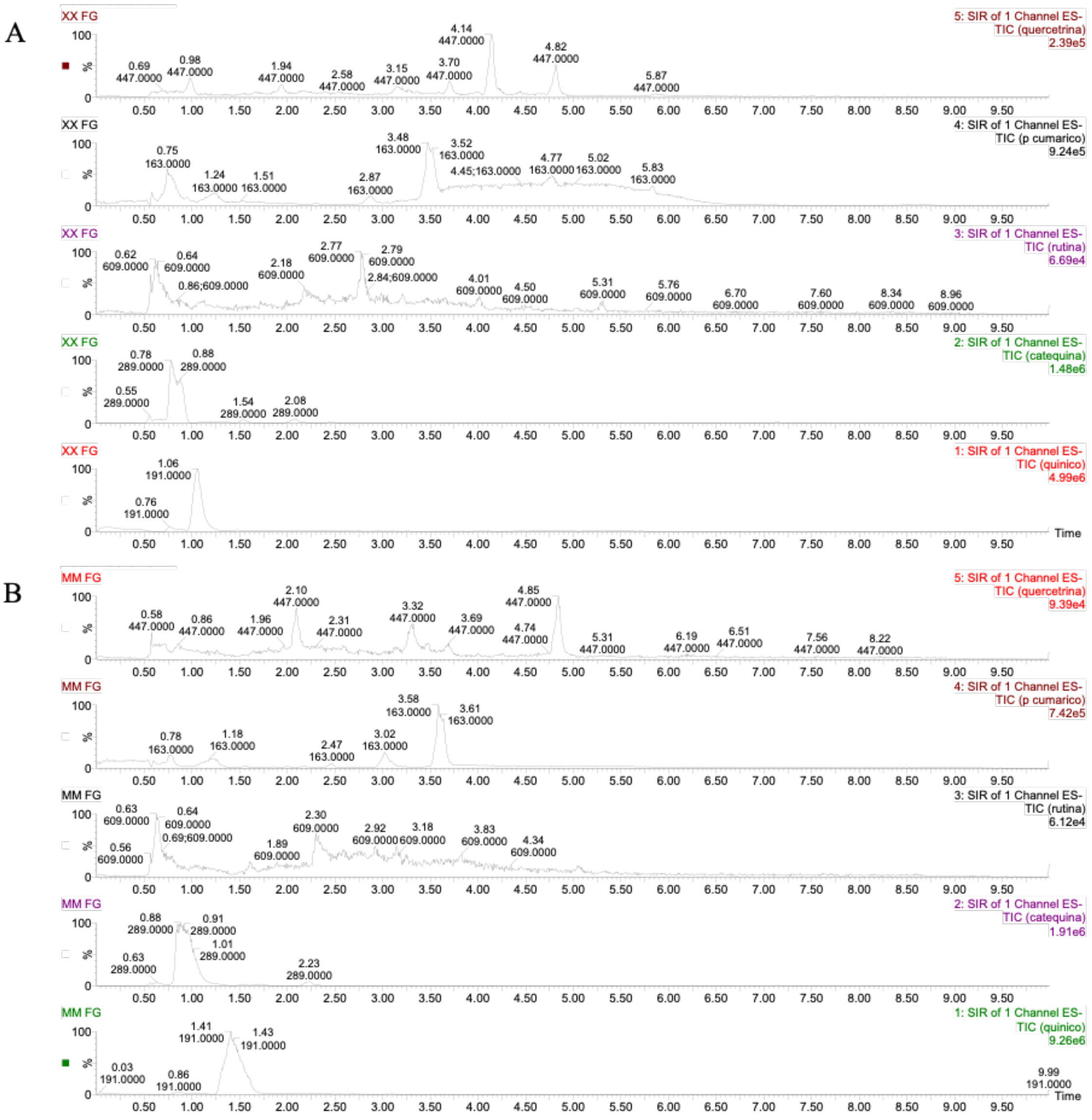

Xique-xique gastric phase (A); *Mandacaru* gastric phase (B).

Figure S3. Chromatogram of samples from the intestinal phases of *xique-xique* and *mandacaru* in LC/MS analysis in Selected Ion Monitoring (SIM) mode.

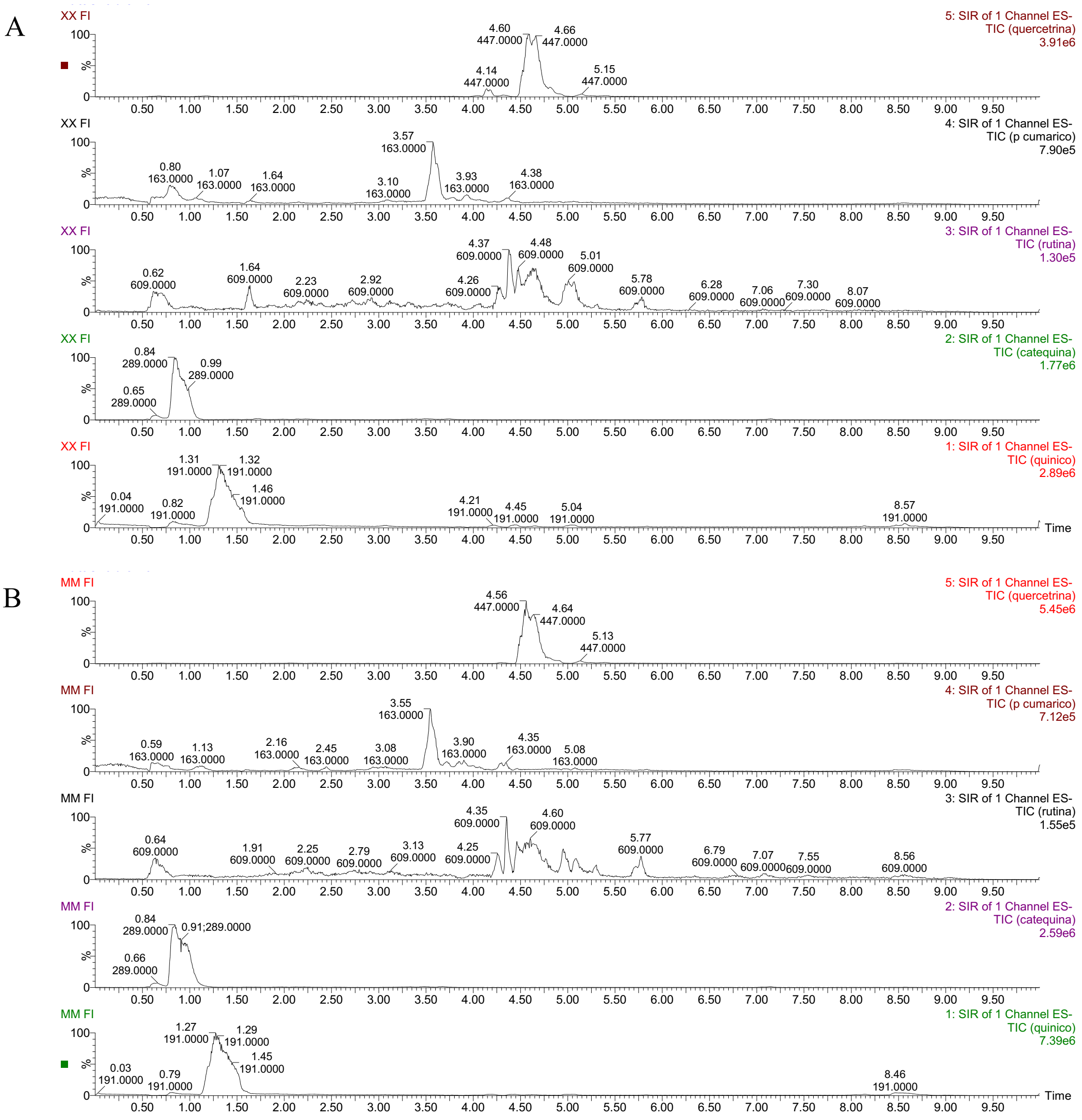

Xique-xique intestinal phase (A); *Mandacaru* intestinal phase (B).
